# Reduction in Overall Time with Corticotomy Using Piezosurgery in Orthodontics: A Meta-Analysis of Randomized Clinical Trials

**DOI:** 10.3390/jcm14061947

**Published:** 2025-03-13

**Authors:** Nur Ebrahim Zaidan, Federico Hernández-Alfaro, Hom-Lay Wang, Jordi Gargallo-Albiol

**Affiliations:** 1 Department of Oral and Maxillofacial Surgery, Universitat Internacional de Catalunya, 08017 Barcelona, Spain; nur01@uic.es (N.E.Z.); h.alfaro@uic.es (F.H.-A.); 2 Department of Periodontics and Oral Medicine, University of Michigan School of Dentistry, Ann Arbor, MI 48109, USA; homlay@umich.edu

**Keywords:** accelerated orthodontics, corticotomy, orthodontic tooth movement, orthodontic treatment, piezocision, treatment time reduction

## Abstract

**Background and Aims:** Decortication of interdental alveolar cortical bone is performed to shorten orthodontic treatment duration. The objective of this meta-analysis was to evaluate the effect of corticotomy using piezosurgery on the reduction in orthodontic treatment time, comparing it to conventional single-phase orthodontic treatment. **Methods:** A thorough electronic search was performed to obtain randomized clinical trials that compared the treatment duration between piezocision-assisted orthodontic treatment with conventional orthodontic treatment. Overall treatment time measured in months, was analyzed, and patient age and tooth location were evaluated as potential factors affecting treatment duration. **Results:** Based on the eleven randomized clinical trials included in the meta-analysis, results showed a significant weighted mean difference between piezocision-assisted corticotomy orthodontics and conventional single-phase orthodontics only (−1.80 months, [95% CI (−2.51, −1.08), *p* = 0.771]). Patient age and tooth location showed no statistically significant difference between the investigated groups. **Conclusions:** Piezocision-assisted corticotomy is effective in accelerating orthodontic tooth movement and reducing an average of 1.80 months treatment time. This reduction, although modest, represents a 30.72% decrease in overall treatment duration.

## 1. Introduction

Corticotomy was introduced into orthodontics as a means of accelerating orthodontic tooth movement (OTM), a process influenced by multiple factors, including optimal force application, turnover in the periodontal ligament, and bone metabolism [1,2]. The primary goal of corticotomy was to shorten treatment duration; however, it has also been shown to mitigate other complications associated with prolonged orthodontic treatments, such as reducing root resorption and improving patient satisfaction. This is due to its ability to facilitate bone block movements rather than individual tooth movements [1].

Initially, corticotomy involved the use of full-thickness flaps and decortication of interdental alveolar cortical bone [3]. This invasive surgical approach potentially induces a regional acceleratory phenomenon (RAP), resulting in a cicatricial response that causes a temporary acceleration of bone metabolism and a decrease in bone density, thereby increasing the rate of bone remodeling and tooth movement, which is beneficial in orthodontic treatments [1,3,4,5,6].

However, the procedure’s invasive nature, particularly the need for mucoperiosteal flap elevation, has led to the development of less invasive techniques. Hence, piezocision was introduced as a means to enhance traditional corticotomy by adopting a less invasive approach [3]. This procedure involves creating interproximal microsurgical openings in the buccal gingiva to facilitate the introduction of a piezoelectric knife, which is then used to decorticate the alveolar bone without elevating large surgical flaps [1,6]. This less invasive alternative to traditional corticotomy eliminates the need for mucoperiosteal flap elevation, reducing surgical time and minimizing postoperative discomfort [5,6]. Additionally, this technique allows for selective tunneling for hard and/or soft tissue grafting if needed [1,5,6].

Despite the growing interest in optimizing orthodontic procedures to decrease treatment time, there is limited consensus on the effectiveness of corticotomy, particularly with newer techniques like piezocision. Several systematic reviews have examined piezocision-assisted orthodontics [7,8,9], but they have heterogeneous methodologies or fail to isolate the effects of piezocision specifically. For example, Apalimova et al. [7] included multiple surgical techniques, resulting in a high level of heterogeneity in both study designs and treatment protocols, without quantifying the treatment time reduction. Although Afzal et al. [8] reported a 46.44% to 95% reduction in treatment duration, their meta-analysis included only four articles, one of which was a non-randomized controlled trial. These limitations often led to inconsistent or inconclusive findings regarding the effectiveness of piezocision, which this meta-analysis aims to address by providing a more reliable and up-to-date analysis focused on piezocision-assisted corticotomy and clarifying its impact on treatment time.

Therefore, the primary aim of this study was to analyze the reduction in orthodontic treatment time when corticotomy using piezosurgery was performed, compared to conventional single-phase orthodontics without corticotomy. The secondary aim was to evaluate patient age and tooth location as potential factors affecting treatment duration.

## 2. Materials and Methods

### 2.1. Study Registration

This review was registered in PROSPERO (International Prospective Register of Systematic Reviews) (University of York, York, UK, YO10 5DD) with the identification number CRD42024507305.

### 2.2. Reporting Format

The 27-item Preferred Reporting Items for Systematic Reviews and Meta-Analyses (PRISMA) statement [10] was used to conduct and finalize the report for each section of this study.

### 2.3. Patient, Intervention, Comparison, Outcome (PICO) Framework

This focused question was performed using the Patient, Intervention, Comparison, Outcome (PICO) standardized approach [11]:

Patient (P): Patients receiving orthodontic treatment.

Intervention (I): Patients receiving orthodontic treatment with piezocision-assisted orthodontic treatment.

Comparison (C): Patients receiving orthodontic treatment without piezocision.

Outcomes (O): Primary outcome: overall treatment time measured in months, and evaluate patient age and tooth location as potential factors affecting treatment duration.

### 2.4. Eligibility Criteria

Articles were included in this systematic review if they met the following criteria: (1) Studies on human subjects; (2) Randomization studies with a minimum of two arms for comparing test and control groups; (3) Piezocision-assisted orthodontic treatment as the test group, compared to orthodontic treatment only as the control group; (4) Reported outcome measures following surgical intervention on the ability of piezocision-assisted orthodontics to reduce overall treatment time. Accordingly, the exclusion criteria consisted of the following: (1) Studies without a comparison group; (2) Animal studies, ex vivo and in vitro studies; (3) Studies lacking measurable clinical outcomes; (4) Lack of objective data for comparing the study group outcome; and (5) Studies lacking overall treatment time with standard deviation.

### 2.5. Information Sources and Search Strategy

An electronic literature search was conducted covering studies from 2015 to 2023, using the National Library of Medicine (MEDLINE by PubMed)(National Library of Medicine, 8600 Rockville Pike, Bethesda, United States) as the primary database search engine, with additional searches in Embase (Elsevier B.V, Radarweg 29 1043 NX Amsterdam, The Netherlands). Using different combinations of the following terms/MeSH/keywords and boolean operators: AND, OR NOT: ((“Piezosurgery”(MeSH) OR piezo*) AND (“Orthodontics”(MeSH) OR orthodontic) AND (“Time Factors”(MeSH) OR treatment time)). The keywords and search strategy can be seen in Table 1 and Table 2, respectively.

Studies were excluded by an initial screening of titles, then by an abstract review, and final screening after full-text reading using the predetermined criteria for inclusion and exclusion previously mentioned.

### 2.6. Data Extraction

The information extracted from each article included the following: (1) authors’ names and year of publication; (2) randomization; (3) test and control group characteristics; (4) setting of the study; (5) follow-up time adjusted in months; (6) comparison protocol; (7) type of orthodontic treatment; (8) extractions; (9) piezosurgery treatment procedure; (10) overall treatment time of test and control groups; (11) rate of orthodontic tooth movement.

### 2.7. Quality Assessment

According to the Cochrane Risk of Bias Tool [12], the potential risk of bias was considered low only if the study provided comprehensive data on all required parameters. Missing one of these parameters was considered to introduce some concern, leading to a classification of moderate risk of bias. If a study lacked two or more of these key parameters, the potential for bias was deemed significant, warranting a high-risk classification. The blinding of participants and personnel was not included in this assessment, as the intervention (piezosurgery) is a surgical procedure, making blinding impractical or impossible.

### 2.8. Statistical Analysis

For evaluating the difference in overall treatment time between piezocision-assisted orthodontics and conventional orthodontics only, two sets of meta-analyses were performed: a meta-analysis to estimate the global effect size in each of the groups (test and control) separately and, a comparative intra-study meta-analysis between the two types of techniques (test and control). Weighted mean differences (WMD) and 95% confidence intervals were calculated using random effects models with the DerSimonian and Laird estimator. The results of the estimates, global effect size, and confidence intervals were represented in a Forest plot. The I^2^ index of heterogeneity was calculated (percentage of variability in the estimated effect that can be attributed to heterogeneity of the true effects), along with the corresponding statistical Q-test for nullity. Publication bias was explored using Funnel plots and Egger’s test. Finally, mixed-effects meta-regression models were estimated, with moderator variables being the location of the treated teeth and the age of the patients. These models investigated whether the hypothetical benefit of piezo-surgery over conventional orthodontics depends on the location of the teeth to be treated or the age of the patients. The significance level used in the analysis was 5% (α = 0.05). The software used for the meta-analysis was R 4.3.2 (R Core Team, 2013. R Foundation for Statistical Computing, Vienna, Austria).

## 3. Results

### 3.1. Study Selection

**Figure 1:**
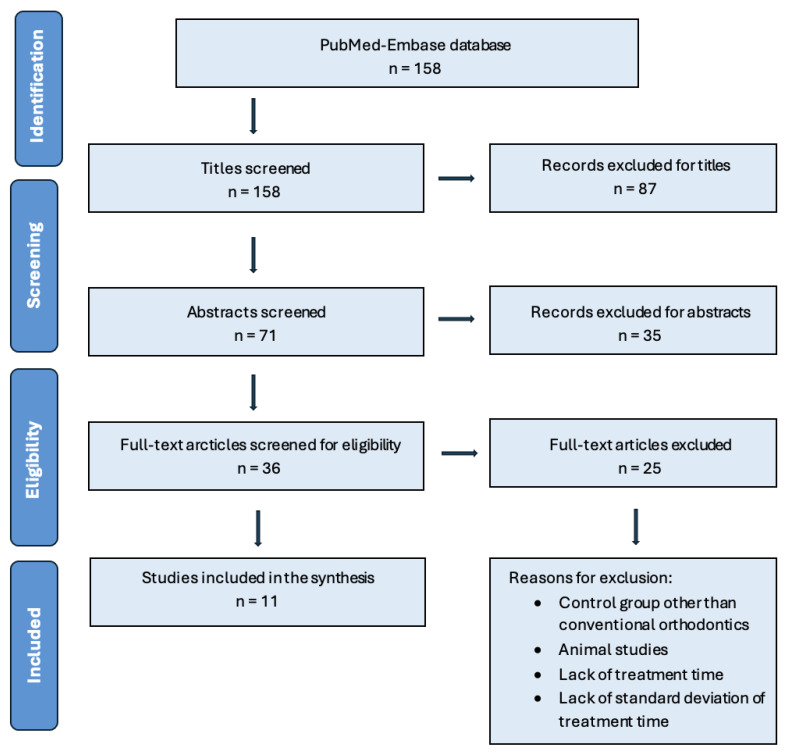
PRISMA flowchart of screening process.

The electronic search yielded 158 articles from the PubMed-Embase database. After the elimination by screening all titles and abstracts, 36 articles remained for full-text assessment. Following a thorough assessment of these articles, 11 RCTs [4,13,14,15,16,17,18,19,20,21,22] were selected for inclusion in this meta-analysis. Details regarding the search, screening process, and exclusion criteria are summarized in Figure 1. The most common reasons for excluding articles were the lack of exact treatment duration for both groups and the absence of standard deviations. The RCT articles were assessed for risk of bias, as shown in the Supplementary Table S1. The overall risk of bias across the included studies was assessed as low, with 55% of articles categorized as having a low risk of bias. However, 18% were rated as moderate and 27% as high risk, indicating variability in methodological quality.

### 3.2. Characteristics of the Included Trials

All RCTs included in this meta-analysis aimed to evaluate the reduction in overall treatment time with piezocision-assisted orthodontics, using orthodontics only as the comparative group, and reported clear outcomes. There was a total of 298 patients, of which 148 received piezocision-assisted corticotomy and 148 received conventional single-phase orthodontics only. Three studies focused on treating severe anterior mandibular crowding [15,18,20], two on severe anterior maxillary crowding [4,22], and the remaining studies addressed bilateral upper canine retraction [17], bilateral upper canine distalization [16], retraction of four upper incisors [13], en masse retraction [14], mild overcrowding [21], and traction of impacted mandibular third molars [19]. Of the 11 studies included, 8 required bilateral first premolar extractions: 6 studies extracted the maxillary first premolars [4,13,16,17,20,22], 1 study extracted the mandibular first premolars [15], and in the study by Tuncer et al. [14], all four first premolars were extracted in cases of Angle Class I malocclusion, while only the maxillary first premolars were extracted in cases of Angle Class II malocclusion. The remaining three studies [18,19,21] did not perform first premolar extractions. A general overview of the included RCTs can be found in Table 3.

### 3.3. Synthesis of Results from Meta-Analysis

The clinical outcomes of the included clinical trials were extracted and organized into tables. The comparison of piezocision-assisted orthodontic treatment with conventional orthodontic treatment was based on eleven articles. The assessment of treatment time reduction, accounting for the effects of piezocision alongside independent factors like tooth location and patient age, was based on all articles [4,13,14,15,16,17,18,19,20,21,22]. The general characteristics of the intervention and results are detailed in Table 4.

Most selected articles have found that piezocision-assisted corticotomy accelerates orthodontic tooth movement [4,13,15,16,17,19,20,21,22]. Contrarily, only two studies [14,18] have found no significant difference when applying piezocision-assisted corticotomy to reduce the overall treatment time in orthodontic treatments.

Regarding the piezoincision technique, the most commonly used measurements range from 3 to 5 mm for piezocision depth or piezocision length [4,13,14,15,16,20,21,22]. However, Alfawal AMH et al. [17] reported a piezocision length of 10 mm, while Uribe et al. [18] described a piezocision depth of 1 mm. In terms of treatment planning and location, severe anterior crowding was the most frequently addressed condition, with three cases in the mandible [15,18,20] and two in the maxilla [4,22], followed by cases of canine malposition [16,17]. A comprehensive overview of the intervention characteristics and outcomes is presented in Table 4.

### 3.4. Piezocision-Assisted Corticotomy vs. Control

**Figure 2:**
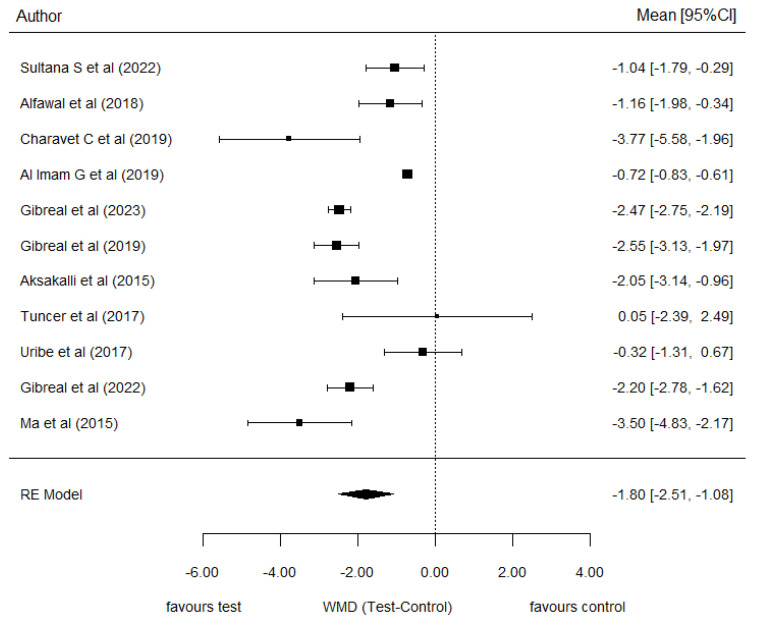
Forest plot of the meta-analysis comparing treatment duration between the piezocision-assisted orthodontics group and the conventional orthodontics group [4,13,14,15,16,17,18,19,20,21,22].

**Figure 3:**
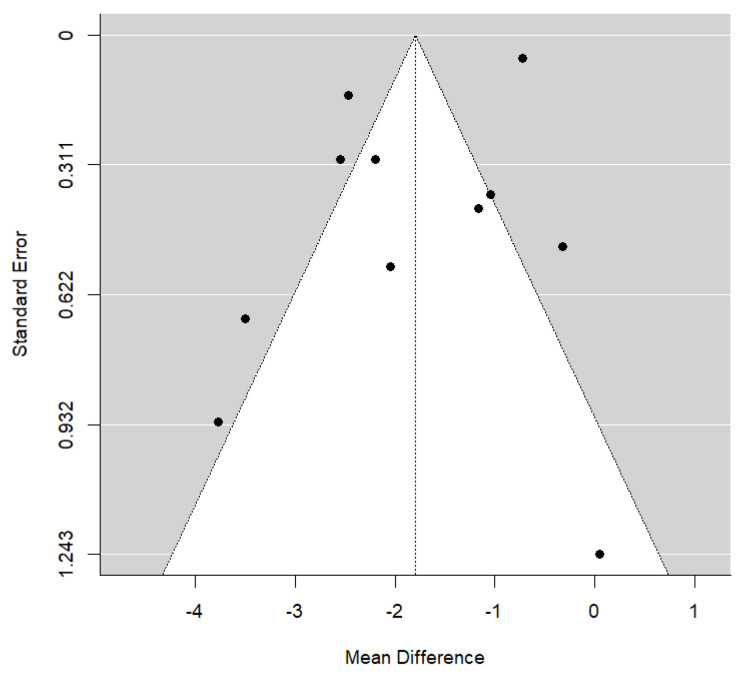
Funnel plot assessing potential publication bias and heterogeneity in the meta-analysis.

Results from the meta-analysis indicated a significant weighted mean difference between piezocision-assisted corticotomy orthodontics and conventional single-phase orthodontics only (−1.80 months, [95% CI (−2.51, −1.08), *p* = 0.771]). The clinical significance of this difference suggests that, on average, patients treated with piezocision completed their treatment approximately 1.80 months earlier than the control group. This reduction represents a 30.72% decrease in overall treatment duration. The forest plot (Figure 2) displays this outcome, and the funnel plot illustrates considerable heterogeneity found in this comparison (I^2^ = 94.9%, *p* < 0.001) (Figure 3). The general symmetry of the plot suggests no significant publication bias, which is further supported by the statistical analysis (*p* = 0.771).

**Table 4:** General Characteristics of the Intervention and Results.

Study (Year)	Treatment	Extractions	Treatment Test Group	Treatment Control Group	Overall Treatment Time (Piezocision Group) (Months)	Overall Treatment Time (Control Group) (months)	Difference in OTT (Control Group is Reference) (Months)	Statistical Significance	Rate of OTM (Distance/Time)	Authors’ Conclusion
Sultana S et al. (2022) [4]	Severe anterior maxillary crowding	First maxillary premolars	Piezocision (PD: 3 mm PL: 4–5 mm)	Orthodontics only	4.04 ± 0.60	5.08 ± 0.72	−1.04	Significantly shorter (20.4% fewer days)	Significantly faster (1.27 times faster)	Piezocision significantly reduces OTM time at the leveling and alignment stage without any adverse effect. The Piezocision group experienced no or mild pain and were satisfied with the treatment
Alfawal AMH et al. (2018) [17]	Canine retraction	First maxillary premolars	Piezocision (PD: 3 mm PL: 10 mm)	Orthodontics only	3.3 ± 0.75	4.49 ± 1.00	−1.19	Significantly shorter (25% reduction in treatment time)	Significantly faster (1.5–2 times faster)	Piezocision accelerates OTM.
Charavet C et al. (2019) [21]	Mild overcrowding	N/A	Piezocision (PD: 3 mm PL: 5 mm)	Orthodontics only	9.11 ± 2.63	12.88 ± 1.83	−3.77	Significantly shorter (36% reduction in treatment time)	Significantly faster (1.6 times faster)	Piezocision accelerates OTM. Scars were observed on 66% of patients.
Al Imam G et al. (2019) [13]	Retraction of four upper incisors	First maxillary premolars	Piezocision (PD: 3 mm)	Orthodontics only	2.02 ± 0.20	2.74 ± 0.16	−0.72	Significantly shorter (27% reduction in treatment time)	Significantly faster (1.53 times faster)	Piezocision accelerates OTM and decreases treatment time.
Gibreal et al. (2023) [22]	Severe anterior maxillary crowding	First maxillary premolars	Piezocision (PD: 3 mm PL: 5–8 mm)	Orthodontics only	2.12 ± 0.38	4.59 ± 0.44	−2.47	Significantly shorter (53% reduction in treatment time)	Significantly faster (2.16 times faster)	Piezocision accelerates OTM.
Gibreal et al. (2019) [15]	Severe anterior mandibular crowding	First mandibular premolars	Piezocision (PD: 3 mm PL: 5–8 mm)	Orthodontics only	1.76 ± 0.08	4.31 ± 1.26	−2.55	Significantly shorter (59% reduction in treatment time)	Significantly faster (2.45 times faster)	Piezocision accelerates OTM in severe crowding cases when accompanied with premolar extractions
Aksakalli et al. (2015) [16]	Bilateral upper canine distalization	First maxillary premolars	Piezocision (PD: 3 mm)	Orthodontics only	3.53 ± 0.81	5.58 ± 0.94	−2.05	Significantly shorter (36% reduction in treatment time)	Significantly faster (2 times faster)	Piezocision accelerates OTM, decreases OTT, is helpful for posterior anchorage control, and does not negatively affect periodontal health.
Tuncer et al. (2017) [14]	En-masse retraction	Class I: All 4 first premolars Class II: First maxillary premolars	Piezocision (PD: 3 mm)	Orthodontics only	9.31 ± 4.09	9.26 ± 2.54	+0.05	Insignificantly longer	Insignificant difference	Piezosurgery showed no significant effect in accelerating en-masse retraction, despite potentially altering tissue reactions.
Uribe et al. (2017) [18]	Severe anterior mandibular crowding	N/A	Piezocision (PD: 1 mm)	Orthodontics only	3.35 ± 1.14	3.67 ± 1.51	−0.32	Insignificantly shorter	Insignificant difference	No treatment time difference between piezocision and conventional orthodontics.
Gibreal et al. (2022) [20]	Severe anterior mandibular crowding	First maxillary premolars	Piezocision (PD: 3 mm PL: 5 mm)	Orthodontics only	2.03 ± 0.41	4.23 ± 1.16	−2.2	Significantly shorter (48% reduction in treatment time)	Significantly faster (2 times faster)	Minimally invasive 3D-guided Piezocision is effective in accelerating OTM.
Ma et al. (2015) [19]	Traction of impacted mandibular third molars	N/A	Piezocision	Orthodontics only	3.99 ± 2.29	7.49 ± 1.30	−3.5	Significantly shorter (46% reduction in treatment time)	Significantly faster (1.87 times faster)	Piezocision accelerates the traction of third molars.

PD: Piezocision depth, PL: Piezocision length, N/A: Not available

### 3.5. Location Impact

As demonstrated by the forest plot, treatment location comparison between the maxilla and mandible indicated a non-significant weighted mean difference favoring the mandible (−0.74 months, [95% CI β (−2.01, 0.52), *p* = 0.249]). The advantage of the piezocision group over the control group in mandibular cases is, on average, 0.74 months greater than in maxillary cases; however, this difference did not reach statistical significance.

### 3.6. Age Impact

**Figure 4:**
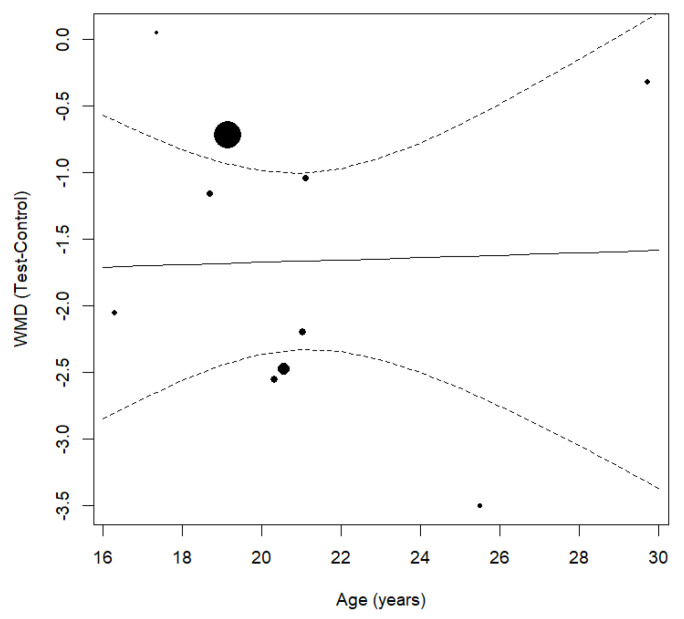
Scatter plot showing the relationship between age and the mean difference in treatment time between the test and control groups.

The comparison of patient age between the investigated groups did not exhibit a statistically significant difference [95% CI β (−0.176, 0.194), *p* = 0.923]). These results are illustrated in Figure 4, which shows a scatter plot with no clear trend between age and treatment time reduction. The data points are spread out with no discernible pattern, further suggesting that age does not significantly influence treatment time reduction in this sample.

## 4. Discussion

This study provides a novel contribution by specifically evaluating corticotomy performed exclusively with piezosurgery, which appears to be the most effective and tissue-friendly technique currently available. Unlike previous studies that compare multiple approaches, our research focuses solely on this method, offering a clearer perspective on its benefits and clinical implications.

The current meta-analysis provides an accurate comparison by strictly controlling the piezocision-assisted corticotomy orthodontics test and control groups and objective clinical outcomes, leading to clear conclusions about the efficacy and advantages of piezocision as an adjunct treatment in orthodontic therapy, focusing on treatment time reduction in piezocision-assisted orthodontics in comparison to conventional orthodontics.

The findings of this meta-analysis revealed that piezocision-assisted corticotomy orthodontics is successful in reducing overall treatment time by 1.80 months in comparison to conventional orthodontic treatment, with 9 out of the 11 included RCTs supporting that piezocision-assisted corticotomy orthodontics yielded to a decrease in treatment time [4,13,15,16,17,19,20,21,22].

Despite the variability in methodology across the studies included in this meta-analysis, with 55% categorized as having a low risk of bias, the majority of studies and the meta-analysis indicate that corticotomy using piezocision is effective in reducing orthodontic treatment time. While heterogeneity in the results exists, the overall trend suggests a positive effect, reinforcing the potential benefits of this technique in orthodontic treatment.

However, it is important to note that the studies by Tuncer et al. [14] and Uribe et al. [18] reported no time reduction related to corticotomy orthodontics. Several factors may contribute to the lack of treatment time reduction despite the use of piezocision-assisted corticotomy, which can directly affect the results of some investigations: (1) inadequate depth or length of the cut, which directly affects RAP; (2) the location of the teeth theoretically plays a role, as maxillary teeth are generally larger, resulting in slower tooth movement in the maxilla compared to the mandible. However, this meta-analysis did not find statistical differences between maxilla and mandible locations; (3) additionally, the frequency of tooth movement activation. Greater movement rates are expected with more frequent activation during the optimal active phase of RAP [4]. In Tuncer et al.’s study [14], where they investigated the impact of piezocision-assisted corticotomy on treatment time in miniscrew-supported en-masse retraction, they found that piezocision-assisted corticotomy did not significantly reduce overall treatment duration. The authors identified that the sampling times were limited, reducing the likelihood of capturing peak molecular changes associated with accelerated bone remodeling. This constraint may have contributed to underestimating the temporary or early biological effects of piezocision-assisted corticotomy on treatment acceleration. Contrarily, the reason why Uribe et al. [18] also reported no reduction in treatment time when alleviating severe anterior mandibular crowding may be attributed to the shallow depth of their piezocision corticotomy cuts, which were only 1 mm deep compared to the standard 3–5 mm. Several studies have reported that 1 mm osteotomy depth is considered insufficient to fully engage the RAP effect, as the effectiveness of RAP on tooth movement is highly dependent on the invasiveness of the procedure [2,23,24]. Another factor that may explain their findings is the deviation from the recommended piezocision-assisted corticotomy protocol, which suggests placing the first arch wire one week prior to surgery and recalling patients every two weeks to sustain the RAP effect [4]. Instead, they placed the wire during surgery and scheduled follow-ups every 4–5 weeks after the initial wire placement.

It is important to recognize that while a reduction of 1.80 months may seem modest, it represents a 30.72% decrease in overall treatment duration, which is clinically relevant. Reducing treatment time is crucial when addressing the risks associated with prolonged treatment durations [13,14,15,17,22]. Future studies could explore whether additional modifications to piezocision protocols might enhance these potential effects.

In relation to tooth location and patient’s age, although treatment location is one of the factors associated with treatment time [4,21], the results did not show a statistically significant difference in treatment time reduction between mandibular and maxillary cases (*p* = 0.249). Only a slight numerical advantage of 0.74 months was observed in favor of the mandible. This trend could be related to differences in bone density between the maxilla and mandible, which may influence the rate of tooth movement. However, the current meta-analysis lacks sufficient statistical power to confirm this hypothesis, highlighting the need for further studies with larger sample sizes and standardized protocols to evaluate the effect of treatment location in greater detail.

Similarly, the patient’s age did not significantly impact treatment time reduction with piezosurgery (*p* = 0.923), as shown in the scatter plot of Figure 4, where the data points display no clear trend, indicating that age did not affect treatment outcomes in this study. This suggests that the biological response to corticotomy may not be heavily age-dependent within the investigated range. However, the lack of an age-based subgroup analysis in many included studies limits the ability to detect potential trends in younger versus older patients. Future research incorporating more detailed categorization by age groups could help clarify whether age-related bone remodeling differences affect piezosurgery outcomes.

Finally, this systematic review and meta-analysis is not exempt from some limitations. Not all existing databases were included, apart from the National Library of Medicine (MEDLINE by PubMed) and Embase, which may have led to the omission of some studies available in other sources. Overall, the number of randomized clinical trials evaluating the use of piezocision in orthodontic treatment was found to be limited. Consequently, the small number of included studies limits the conclusions that can be drawn. While other types of studies, such as non-randomized clinical trials, retrospective studies, and case series, are abundant, including them would compromise the quality of the meta-analysis and hinder the ability to reach clear and objective scientific conclusions. In addition, orthodontic treatment acceleration may be affected by various factors, such as the treatment plan, movement strategy, location, and decisions related to extractions. Despite this, a sample size of studies meeting the inclusion criteria and, above all, the randomization, conditioned the analysis. Different treatment subgroup modalities could have underpowered the study, limiting its ability to assess the effectiveness of piezoincision-assisted corticotomy in reducing orthodontic treatment time.

Therefore, further studies with larger sample sizes and well-defined outcome measures are needed, with careful consideration of treatment location, treatment plan, and patient age.

## 5. Conclusions

The findings of this meta-analysis suggest that corticotomy using piezocision is effective in accelerating orthodontic tooth movement and reducing treatment time. Additionally, factors like tooth location and patient age did not show a clear trend in affecting the treatment duration. Future studies should aim to investigate the impact of these factors more thoroughly, utilizing larger sample sizes and more controlled conditions to better understand the variables that may influence the effectiveness of piezocision.

## Figures and Tables

**Table 1:** Keywords.

Keywords
Accelerated orthodontics
Corticotomy
Orthodontic tooth movement
Orthodontic treatment
Piezocision
Treatment time reduction

**Table 2:** The Search Strategy.

Search Term	PubMed Search Strategy	Embase Search Strategy
Piezosurgery	(“Piezosurgery”[MeSH] OR piezo*)	‘piezosurgery’ OR ‘piezo*’
Orthodontics	(“Orthodontics”[MeSH] OR orthodontic)	‘orthodontics’ OR ‘orthodontic’
Time Factors	(“Time Factors”[MeSH] OR treatment time)	‘time factors’ OR ‘treatment time’
Combined Strategy	((“Piezosurgery”[MeSH] OR piezo*) AND (“Orthodontics”[MeSH] OR orthodontic) AND (“Time Factors”[MeSH] OR treatment time))	(‘piezosurgery’ OR ‘piezo*’) AND (‘orthodontics’ OR ‘orthodontic’) AND (‘time factors’ OR ‘treatment time’)

**Table 3:** General Overview of the Included Studies.

Study (Year)	Study Design	Range, Mean Age	*n* (Patients)	Follow-Up	Comparison Protocols	Location	Setting
Sultana S et al. (2022) [4]	RCT	21.07, SD ± 2.69 (18–30)	13	Before treatment, 1 and 2 months post-treatment, and at the end of leveling and alignment stage	Piezocision	Maxillary	The Orthodontics Unit of University Sains Malaysia, Malaysia
Alfawal AMH et al. (2018) [17]	RCT	18.70, SD ± 3.6	18	Two-week interval and 1-, 2-, 3-, and 4-month measurements taken	Piezocision	Maxillary	Orthodontic Department of the University of Damascus Dental School, Syria
Charavet C et al. (2019) [21]	RCT	27.90, SD ± 7.6	24	Every 2 weeks, archwires were changed only when full bracket engagement was achieved	Piezocision	Maxillary and mandibular	University Hospital Liege, Belgium
Al Imam G et al. (2019) [13]	RCT	19.15, SD ± 3.40	42	Maxillary alginate impressions taken at the onset and every 3 weeks until week 12. Cephalometry at onset and week 12	Piezocision	Maxillary	Department of Orthodontics at the University of Damascus Dental School, Syria
Gibreal et al. (2023) [22]	RCT	20.56, SD ± 3.71	32	Wire changes made when necessary, until full alignment was achieved	Piezocision	Maxillary	Department of Orthodontics at the University of Damascus Dental School, Syria
Gibreal et al. (2019) [15]	RCT	20.32, SD ± 1.96	36	Two-week interval. Little’s Irregularity Index (LII) was calculated at monthly intervals	Piezocision	Mandibular	Departments of Oral and Maxillofacial Surgery and Orthodontics at the University of Damascus Dental School, Syria
Aksakalli et al. (2015) [16]	RCT	16.30, SD ± 2.4	10	Two-week interval. Pre- and post-distalization model casts	Piezocision	Maxillary	Department of Orthodontics, Faculty of Dentistry, Bezmialem Vakif University, Istanbul, Turkey
Tuncer et al. (2017) [14]	RCT	17.35, SD ± 2.6	30	Rates were measured on days 15, 30, 60, 90, and 120. Dental casts were obtained before and after treatment	Piezocision	Maxillary	Department of Orthodontics, Faculty of Dentistry, Baskent University Ankara, Turkey
Uribe et al. (2017) [18]	RCT	29.73, SD ± 11.19	29	Experimental subjects were monitored 1 week post-surgery. All subjects were followed monthly after the first wire placement, with mandibular study casts taken every 4–5 weeks	Piezocision	Mandibular	Division of Orthodontics, Department of Craniofacial Sciences, University of Connecticut School of Dental Medicine, Farmington, USA
Gibreal et al. (2022) [20]	RCT	21.03, SD ± 1.96	34	Two-week interval. Little’s Irregularity Index (LII) was calculated using dental casts before, 1 month, 2 months, and after treatment	Piezocision	Mandibular	Department of Orthodontics at the University of Damascus Dental School, Syria
Ma et al. (2015) [19]	RCT	25.50, SD ± 5.2	30	Forty-eight h post-surgery. Monthly monitoring after the orthodontic appliance was inserted	Piezocision	Mandibular	Department of Oral Surgery, Shanghai Ninth People’s Hospital affiliated with Shanghai Jiao Tong University, School of Medicine, Shanghai, China

## Data Availability

The original contributions presented in this study are included in the article/Supplementary Materials. Further inquiries can be directed to the corresponding author.

## Supplementary Materials

The following supporting information can be downloaded at: https://www.mdpi.com/article/10.3390/jcm14061947/s1, Supplementary Table S1: Bias Risk Assessment for the Included RCTs Using the Cochrane Risk of Bias Tool for Randomized Controlled Trials.

## Abbreviations

OTM	orthodontic tooth movement
RAP	regional acceleratory phenomenon
