# Supplementary material for: Reduction in Overall Time with Corticotomy Using Piezosurgery in Orthodontics: A Meta-Analysis of Randomized Clinical Trials
Source: J Clin Med. 2025 Mar 13;14(6):1947. doi: 10.3390/jcm14061947 (PMC11943040; doi:10.3390/jcm14061947)
Supplement: Supplementary file 1 [file jcm-14-01947-s001.zip › jcm-3497762-supplementary.pdf]

Supplementary Materials

Supplemental Table S1	Bias Risk Assessment for the Included RCTs Using the Cochrane Risk of Bias Tool for Randomized Controlled Trials							
**Study**	**Random Sequence generation**	**Allocation concealment**	**Blinding of participants and personnel**	**Blinding of outcome assesment**	**Incomplete outcome data**	**Selective reporting**	**Other bias**	**Overall risk of bias**
Sultana S et al[4], 2022	low	low	high	moderate	low	low	low	moderate
Alfawal AMH et al[17], 2018	low	low	high	low	low	low	low	low
Charavet C et al[21], 2019	low	moderate	moderate	low	low	low	low	moderate
Al Imam G et al[13], 2019	low	low	high	low	low	low	low	low
Gibreal et al[22], 2023	low	low	high	low	low	low	low	low
Gibreal et al[15], 2019	low	low	high	low	low	low	low	low
Aksakalli et al[16], 2015	low	high	moderate	moderate	low	low	low	high
Tuncer et al[14], 2017	moderate	low	high	low	low	low	low	high
Uribe et al[18], 2017	low	low	moderate	low	low	low	low	low
Gibreal et al[20], 2022	low	low	moderate	low	low	low	low	low
Ma et al[19], 2015	low	high	high	high	low	low	low	high
